# Detection of Fusobacterium Nucleatum and fadA Adhesin Gene in Patients with Orthodontic Gingivitis and Non-Orthodontic Periodontal Inflammation

**DOI:** 10.1371/journal.pone.0085280

**Published:** 2014-01-09

**Authors:** Ping Liu, Yi Liu, Jianning Wang, Yang Guo, Yujie Zhang, Shuiqing Xiao

**Affiliations:** 1 Department of Orthodontics, Jinan Stomatological Hospital, Jinan, China; 2 Pediatric Research Institute, Qilu Children's Hospital of Shandong University, Ji'nan, China; 3 Department of Periodontics, Jinan Stomatological Hospital, Jinan, China; University of Missouri-Kansas City, United States of America

## Abstract

*Fusobacterium nucleatum* is one of the most abundant gram-negative bacilli colonizing the subgingival plaque and closely associated with periodontal disease. However it is unclear whether *F. nucleatum* is involved in gingival inflammation under orthodontic appliance. A novel adhesin, *FadA*, which is unique to oral *Fusobacteria*, is required for *F. nucleatum* binding and invasion to epithelial cells and thus may play an important role in colonization of *Fusobacterium* in the host. In this study, we evaluated the prevalence of *F. nucleatum* and its virulence factor *FadA* adhesion gene (*fadA*) in 169 subgingival biofilm samples from 55 cases of gingivitis patients with orthodontic appliances, 49 cases of gingivitis patients without orthodontic treatment, 35 cases of periodontitis patients and 30 cases of periodontally healthy people via PCR. The correlations between the *F. nucleatum*/*fadA* and gingivitis index(GI)was also analyzed. The detection rate of *F. nucleatum*/*fadA* in periodontitis group and non-orthodontic gingivitis group was higher than the other two groups (p<0.01) while it was higher in orthodontic gingivitis group than in health people (p<0.05). An obviously positive correlation was observed between the prevalence of *F. nucleatum*/*fadA* and GI. *F. nucleatum* carrying *fadA* may be more closely related to the development of gingivitis and periodontal disease compared with orthodontic gingivitis.

## Introduction

Fixed orthodontic treatment is currently the preferred and most common method for malocclusion which is a frequently-occurring disease affecting facial appearance and chewing function. During orthodontic therapy, orthodontists are frequently confronted with gingivitis [1]. Studies have reported that orthodontic attachments can accelerate the accumulation of bacterial plaque for the difficulties in maintaining oral hygiene [2]. Also the placement of orthodontic appliances affects the subgingival microbial composition even during the early period of orthodontic treatment, increasing the prevalence of periodontopathogens [2].

*F. nucleatum* is a gram-negative anaerobes ubiquitous in the oral cavity, presenting in both healthy and diseased periodontal sites and associated with various forms of periodontal diseases [3]. The bacterium has been reported to induce apoptosis in gingival epithelial cells and polymorphonuclear blood cells; in addition, it suppresses immunological defense mechanisms [4], [5] and induces innate immune responses [6], [7].A novel adhesin, *FadA*, was

identified to be involved in *F. nucleatum* attachment and invasion to host cells and highly conservative among oral *Fusobacteria* species [8]. Previous studies have shown that *F. nucleatum* is closely related to adult and juvenile periodontitis [9]-[11], but little researches on gingival response to *F. nucleatum* and its virulence factor *FadA* adhesin during orthodontics.

Our previous research has showed that *Porphyromonas gingivalis*, the gram-negative oral anaerobe, is one of the risk factors that are responsible for orthodontic gingivitis and periodontitis[12]. However, another periodontitis-associated bacterium, *F. nucleatum* with little available information has not been detected. The purpose of this study was to evaluate the prevalence of *F. nucleatum* and *FadA* adhesin in subgingival biofilm samples from the gingivitis lesions of orthodontic patients and compared them with that of non-orthodontic gingivitis and periodontitis patients as well as periodontal healthy people who showed healthy periodontal tissues before wearing orthodontic appliances. Also, the correlation between detection rate of *F. nucleatum*/*fadA* and GI was analyzed.

## Materials and Methods

### Subjects

The study subjects consisted of four groups who visited Jinan Stomatological Hospital for orthodontics or periodontitis treatment from 2011 to 2013. Of four groups, orthodontic group (OG) included 55 patients, 21 females and 34 males, aged between 11 and 27 years (mean 16.25) who got gingival inflammation during orthodontic treatment; control group (CG) contained 30 periodontal healthy people, 18 females and 12 males, aged between 12 and 26 years (mean 19.40) before orthodontic treatment; non-orthodontic gingivitis group (NOG) was made up of 49 gingivitis patients without orthodontic treatment, 26 females and 23 males, aged between 12 and 25 years(mean 16.62); periodontitis group (PG) was composed of 35 periodontitis patients, 16 females and 19 males, aged from 22 to 68 years (mean 46.46). These patients with any systemic diseases, antibiotics therapy within the last 3 months and pregnant or lactating females were excluded.

### Ethics statement

This work was approved by the Medical Ethics Committee of the Jinan Stomatological Hospital. We obtained written informed consents from the patients or parents on the behalf of all children participants involved in the study before the examination was performed. The relevant regulations and institutional polices were followed strictly.

### Bacteria strains

The reference strains of *F.nucleatum* ATCC25586 and *Aggregatibacter actinomycetemcomitans* ATCC29522 were from the West-China Dental School of Si Chuan University. *Porphyromonas gingivalis* W83 and *Streptococcus mutans* ATCC25175 were from Beijing Oral Research Institute of Capital Medical University.

### Evaluation of gingival status

According to the standard revised by Loe [13], gingival status was checked and recorded in four gums areas: buccal gingival papilla, mesial buccal marginal gingiva, buccal and distal gingival papilla, lingual marginal gingiva. Gingival inflammation was divided into three levels, 0, 1, 2 and gingival index (GI) was assessed. All clinical examinations were carried out by the same dentist.

### Sample collection and DNA extraction

Subgingival biofilm was obtained from the deepest periodontal pockets as described before [14], [15] In brief, before collecting, saline solution was used to rinse out food debris and then each site was cleaned by cotton rolls. Visible supragingival plaque was removed. A sterile paper point was inserted into the pocket for 30 seconds until a minimum of resistance was felt. The paper point was immediately transferred into a sterile microcentrifuge tube containing 0.5 ml of 1×PBS. The tubes were mixed thoroughly and stored at −20°C until analyzed. The bacterial DNA was extracted by the boiling method [12], [16]. In short, a 10 µl aliquot of each stored sample was added to 10 µl of 2 ×lysis buffer (2 mM EDTA, 1% X-100). The mixture was boiled for 10 minutes and then placed on ice. The supernatant was used as the template for PCR amplification.

### Specificity of the 16S rRNA-based PCR

Specificity of the 16S rRNA-based PCR was evaluated by using specific primers of 16SrRNA gene and the reference strains, including *F.nucleatum* ATCC25586, *A. actinomycetemcomitans* ATCC29522, *P. gingivlis* ATCC33277 and *S. mutans* ATCC25175l. The amplified products from clinical samples were randomly chosen for sequencing.

### The 16S rRNA-based PCR and *FadA* specific PCR

The 16S rRNA-based PCR was used to determine the prevalence of *F. nucleatum* in subgingival biofilm. The PCR was performed on DNA extracts from subgingival biofilm samples using *F. nucleatum* primers of 16S rRNA-F (5′-AGA GTT TGA TCC TGG CTC AG -3′) and 16S rRNA-R (5′-GTC ATC GTG CAC ACA GAA TTG CTG-3′) to amplify a 360-bp region of the 16S rRNA gene[17], while using *fadA* primers of *fadA*-F (5′-CAC AAG CTG ACG CTG CTA GA -3′) and *fadA*-R (5′-TTA CCA GCT CTT AAA GCT TG -3′) to amplify a 232-bp region of the *FadA* gene (designed for this study) from positive samples of *F.nucleatum*. Amplification reaction was run in a Tetrad Thermal Cycler (MJ Research, South San Francisco, USA) in a 25 µl reaction mixture containing 4.5 µl 10×PCR buffer (100 mM Tris-HCl, 500 mM KCl, and 15 mM MgCl2), 0.25 mM of each deoxynucleoside triphosphate (dNTP), 10 µM of each primers, 5 µl of DNA extracts from subgingival biofilm samples, and 1.5 units of Taq DNA polymerase (Transgen Biotech, Beijing). The 16S rRNA PCR of *F. nucleatum* was carried out for 5 min at 94°C and 30 cycles, with each cycle consisting of denaturation at 94°C for 30 sec, annealing at 58°C for 30 sec, extension at 72°C for 1 min, and final extension for 10 min. The PCR of *fadA* was carried out for 4 min at 94°C and 30 cycles, with each cycle consisting of denaturation at 94°C for 30 sec, annealing at 55.8°C for 30 sec, extension at 72°C for 40 sec, and final extension for 6 min.

The amplified products were then electrophoresed on 1.5% agarose gel in Tris-acetate buffer (40 mM Tris acetate, 1 mM EDTA, pH8.0). The products were visualized with ethidium bromide by UV transillumination.

### Statistical analysis

Chi-squared test was used to compare detection rates of *F. nucleatum* and *fadA* among four groups. The Spearman's rank correlation analysis was utilized to determine the correlation between prevalence of *F. nucleatum*/*fadA* genes and GI in four research groups. All statistical analyses were done by using a statistical software package (SPSS for Windows 17.0). p<0.05 were considered to be statistically significant.

## Results

### Detection and confirmation of 16S rRNA-based PCR for *F. nucleatum*

The reference strains were first amplified by the 16S rRNA-based PCR to evaluate the specificity of it. Agarose gel electrophoresis showed that a 360bp specific amplification was obtained only from *F.nucleatum* ATCC25586, not from *P.gingivalis* W83, *A.actinomycetemcomitans* ATCC29522, *S.mutans* ATCC25175 and double distilled water.

**Figure 1:**
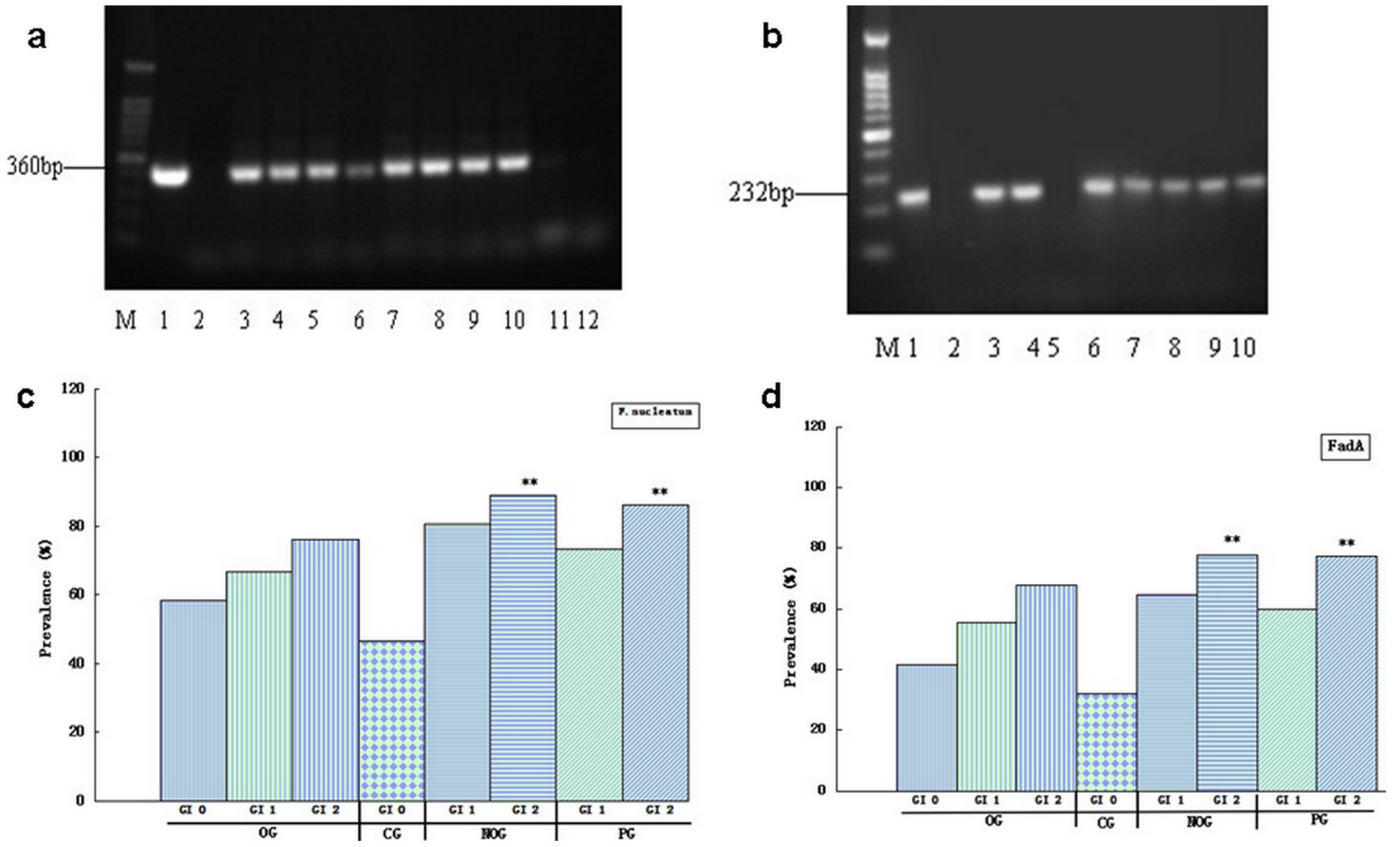
Detection and distribution of *F. nucleatum/fadA*. a. Detection of *F. nucleatum* in clinical subgingival biofilm samples. M:Marker;lane 1:positive control of *F. nucleatum* ATCC25586;lane 2:blank; lane 3∼10:positive clinical samples;lane 11 and12:negative clinical samples; b. Detection of *fadA* in clinical subgingival biofilm samples. M:Marker;lane 1:positive control of *F. nucleatum* ATCC25586;lane 2:blank;lane 3,4,6∼10:positive clinical samples;lane 5: negative clinical sample; c. Distribution of *F. nucleatum* in four groups. d. Distribution of *fad*A in four groups. ** P<0.01 between GI 2 and GI 0 in PG and NOG (c, d) (Chi-squared test).

*F. nucleatum* was detected in 122 (72.19%) cases of subgingival biofilm samples from 169 cases of four groups, 38 (69.09%) from OG, 14 (46.67%) from CG, 41(83.67%) from NOG, and 29 (82.86%) from PG (Fig 1.a, Table 1).

**Table 1:** Prevalence of *F. nucleatum* and *fadA* among four groups.

Groups	Cases(n)	*F. nucleatum*		*fadA*	
		counts	Detection rate(%)	counts	Detection rate(%)
CG	30	14	46.67	10	33.33
OG	55	38	69.09*	32	58.18*
NOG	49	41	83.67**	34	69.39**
PG	35	29	82.86**	25	71.43**
Total	169	122	72.19	101	59.76

P<0.05 between OG/NOG/PG and CG; **P<0.01 between OG/NOG/PG and CG (Chi-squared test).

**Figure 2:**
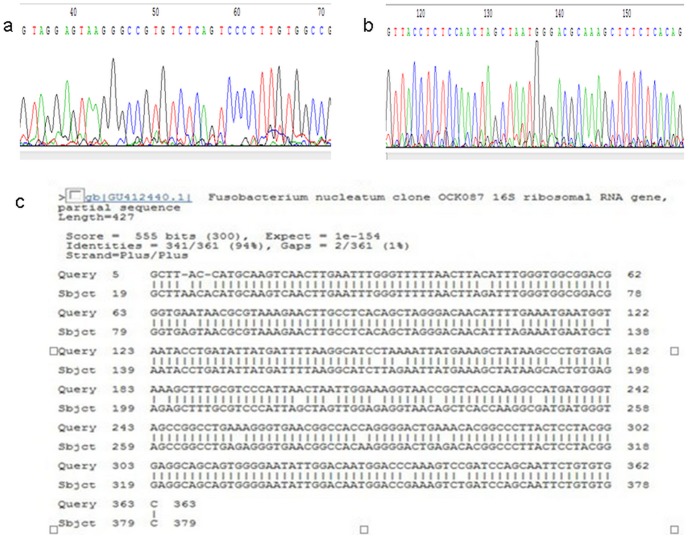
Sequencing chromatograms from F. nucleatum PCR product. a. F primer; b. R primer; c. DNA sequencing and BLAST analysis results.

Ten out of 122 *F. nucleatum* positive samples were randomly selected for sequencing in Invitrogen Company (Invitrogen, Shanghai) to confirm the validity of the 16S rRNA-based PCR in clinical subgingival biofilm samples (Fig. 2).

### PCR amplification of *FadA* gene

*fadA* specific PCR was used to amplify *FadA* gene firstly from the reference strains of *F.nucleatum* ATCC25586 and then from 122 positive samples of *F. nucleatum* to generate a 232-bp product.

There were 101 *fadA* positive samples when *fadA* primers were used to amplify *FadA* gene from 122 *F.nucleatum* positive samples. The detection rate of *fadA* in all cases of subgingival samples from four groups was 59.79%, 58.18% from OG, 33.33% from CG 69.39% from NOG, and 71.43% from PG, individually (Fig 1.b, Table 1).

For both of *F. nucleatum* and *fadA*, the detection rate was higher in group OG than that in group CG(P<0.05); while the detection rates in group PG and NOG were significantly higher than that in group CG(P<0.01)(Table 1).

### Correlation of *F. nucleatum*/*fadA* and GI

We found that the prevalence of *F nucleatum* and *fadA* increased with GI value. For detection of *F. nucleatum*, 20 out of 40 (50%) cases in level 0 of GI were positive; in level 1 of GI, 48 (75%) were positive and 54 (80.85%) were positive in level 2 of GI; For *fadA*, in GI 0, 14 out of 40(35%) were positive; in GI 1, 39 (60.94%) were positive and in GI 2, 48 (73.85%) were positive. From 122 positive cases for *F. nucleatum*, 101(82.79%) were also positive for *fadA*. The detection rates of *F. nucleatum* and *fadA* rose with GI in clinical samples. An obvious positive correlation(P<0.05)was observed between GI and the prevalence of *F. nucleatum*/*fadA* by using Spearman's rank correlation analysis. However, there was no statistical difference between positive rate of *F.nucleatum*/*fadA* and GI in OG. The prevalence of *F.nucleatum*/*fadA* was observed significantly higher only in GI 2 from PG and NOG than from CG (Fig.1c, 1d).

## Discussion

Sallum et al. [17] investigated the clinical and microbiologic changes after removal of orthodontic appliances and found periodontal pathogens such as *A.actinomycetemcomitans* and *B.forsythus* were associated with gingival inflammation during orthodontic treatment. *F. nucleatum* is reported playing an important role for periodontal diseases [3], [18]. In this study, we detected prevalence of *F. nucleatum* and *FadA* adhesin gene in subgingival biofilm in local patients of orthodontic gingivitis, non-orthodontic gingivitis, periodontitis as well as periodontally healthy people to evaluate the distribution of *F. nucleatum* and *fadA* in different periodontal health status, then further deduced the pathogenicity of *F. nucleatum* carrying *fadA*.

We randomly collected subgingival biofilm samples with sterile paper point from 169 patients. The prevalence of *F. nucleatum* was detected and the correlation of it with GI was analyzed. There were significant differences among the four group(P<0.01). Meanwhile there was a positive correlation between the positive rate and GI by using Spearman's rank correlation analysis. The detection rate of *F. nucleatum* as one of main periodontal inflammation pathogens increased with the severity of periodontal lesion. However, there were no statistical differences among positive rates of *F. nucleatum* in three GI levels in OG, while detection rate of *F. nucleatum* in GI2 from PG and NOG was significantly higher than that from both groups of CG and OG. After wearing the fixed appliance, such as brackets, bands and arch wires, the accumulation of bacterial plaque increases the difficulty of maintaining oral hygiene, which may result in increased sulcus bleeding index, gingival inflammation and hyperplasia [19]–[22]. Orthodontic treatment may create a living environment more conducive to periodontal anaerobe such as *F. nucleatum*, which might imply a potential risk for periodontal health in certain patients after longtime orthodontic treatment.

Some relevant clinical studies confirmed the differences between orthodontic gingivitis and periodontitis. Polson et al. [23] found that orthodontic treatment during adolescence had no distinct effect upon later periodontal health. Gingival inflammation and gingival bleeding will increase in teenagers as a result of the hormone changes that occur during puberty [24]. A systematic review identified an absence of reliable evidence describing positive effects of orthodontic treatment on periodontal health, but many findings indicated that orthodontic therapy resulted in small detrimental effects to the periodontium [24]. A controlled clinical study of persons who had completed orthodontic therapy at least 10 years previously compared to a group of adults with untreated malocclusion demonstrated that orthodontic treatment during adolescence had no distinct effect upon later periodontal health [23]. In this study, we analyzed correlation of patients' age and occurrence of *F. nucleatum* and found the age of both *F. nucleatum* positive and negative was statistically different which implied the prevalence of *F. nucleatum* may increase with patients'age, while the incidence of periodontal disease also increases. Therefore, longitudinal studies including large amount of samples are required to find the impact of *F. nucleatum* colonization on periodontal conditions during and after orthodontic therapy.

Bacterial adhesion is usually the first step for a periodontal pathogen to infect and invade the host cells. In 2005, a novel adhesin, *FadA*, which is unique to oral *Fusobacteria* was identified by Han et al [8]. It was required for *F. nucleatum* to attach epithelial cells and thus may play an important role in *Fusobacterium* colonization in the host. In this study, we further detected the distribution of *fadA* in four groups to investigate whether it is involved in

gingival inflammation under orthodontic appliance. The detection rate of *fadA* decreased in turn from NOG, PG, OG to CG group. Also, it had an upward trend with the increase of gingival index. A clear positive correlation was indicated between GI and *FadA* gene by using Spearman's rank correlation analysis. However, only the prevalence of *fadA* in GI 2 from PG and NOG was significantly higher than that in CG, while there were no statistical difference among positive rate of *fadA* in three GI levels in OG. The *F. nucleatum* carrying *fadA* may have a higher pathogenicity and could lead to a classification of these strains, which is more closely related to the development of non-orthodontic periodontal inflammation rather than gum inflammation during orthodontic treatment. On the contrary, the *F. nucleatum* without

*fadA* may represent the avirulent or weak virulence genotype of *F. nucleatum*.

In summary, *F. nucleatum* carrying *fadA* is one of the potential risks that are responsible for non-orthodontic periodontal inflammation. All orthodontic patients must receive oral hygiene instruction and professional prophylaxis to maintain gingival health. Moreover, further research is needed to verify the periodontal potential health risks and to find the most effective way of controlling periodontal pathogenic anaerobic bacteria during orthodontic treatment.
